# ThyroidPrint®: clinical utility for indeterminate thyroid cytology

**DOI:** 10.1530/ERC-22-0409

**Published:** 2023-10-06

**Authors:** Roberto Olmos, José Miguel Domínguez, Sergio Vargas-Salas, Lorena Mosso, Carlos E Fardella, Gilberto González, René Baudrand, Francisco Guarda, Felipe Valenzuela, Eugenio Arteaga, Pablo Forenzano, Flavia Nilo, Nicole Lustig, Alejandra Martínez, José M López, Francisco Cruz, Soledad Loyola, Augusto Leon, Nicolás Droppelmann, Pablo Montero, Francisco Domínguez, Mauricio Camus, Antonieta Solar, Pablo Zoroquiain, Juan Carlos Roa, Estefanía Muñoz, Elsa Bruce, Rossio Gajardo, Giovanna Miranda, Francisco Riquelme, Natalia Mena, Hernán E González

**Affiliations:** 1 Department of Endocrinology, School of Medicine Pontificia Universidad Católica de Chile; 2 Department of Surgical Oncology, School of Medicine Pontificia Universidad Católica de Chile; 3 Department of Radiology, School of Medicine Pontificia Universidad Católica de Chile; 4 Department of Anatomic Pathology, School of Medicine Pontificia Universidad Católica de Chile

**Keywords:** indeterminate thyroid cytology, genetic classifier, clinical utility

## Abstract

Molecular testing contributes to improving the diagnosis of indeterminate thyroid nodules (ITNs). ThyroidPrint® is a ten-gene classifier aimed to rule out malignancy in ITN. Post-validation studies are necessary to determine the real-world clinical benefit of ThyroidPrint® in patients with ITN. A single-center, prospective, noninterventional clinical utility study was performed, analyzing the impact of ThyroidPrint® in the physicians’ clinical decisions for ITN. Demographics, nodule characteristics, benign call rates (BCRs), and surgical outcomes were measured. Histopathological data were collected from surgical biopsies of resected nodules. Of 1272 fine-needle aspirations, 109 (8.6%) were Bethesda III and 135 (10.6%) were Bethesda IV. Molecular testing was performed in 155 of 244 ITN (63.5%), of which 104 were classified as benign (BCR of 67.1%). After a median follow-up of 15 months, 103 of 104 (99.0%) patients with a benign ThyroidPrint® remained under surveillance and one patient underwent surgery which was a follicular adenoma. Surgery was performed in all 51 patients with a suspicious for malignancy as per ThyroidPrint® result and in 56 patients who did not undergo testing, with a rate of malignancy of 70.6% and 32.1%, respectively. A higher BCR was observed in follicular lesion of undetermined significance (87%) compared to atypia of undetermined significance (58%) (*P* < 0.05). False-positive cases included four benign follicular nodules and six follicular and four oncocytic adenomas. Our results show that, physicians chose active surveillance instead of diagnostic surgery in all patients with a benign ThyroidPrint® result, reducing the need for diagnostic surgery in 67% of patients with preoperative diagnosis of ITN.

## Introduction

Indeterminate thyroid nodules (ITNs) account for nearly 20% of thyroid cytology reports. Additionally, the extensive availability of routine high-resolution ultrasound and fine-needle aspiration (FNA) biopsies performed to study thyroid nodules has resulted in an increasing number of ITN during the last years (Haugen *et al.* 2016, Grani *et al.* 2020). Recently, molecular testing has been proposed as part of the diagnostic algorithm by the American Thyroid Association (ATA) guidelines, resulting in a meaningful reduction of diagnostic surgeries (Koster *et al.* 2018, Vargas-Salas *et al.* 2018, Carty *et al.* 2020). Commercially available molecular tests for ITN are based on multi-analyte assays, either by identifying genetic abnormalities by next-generation sequencing and gene expression (Afirma GSC, Thyroseq V3, ThyrGeNext) or miRNA expression profiles by quantitative PCR (q-PCR) (ThyroidPrint, MirThype) (Gonzalez *et at.* 2017, Dos Santos *et al.* 2018, Nikiforova *et al.* 2018, Patel *et al.* 2018, Lupo *et al.* 2020). The primary intended use of these tests is to predict benign thyroid nodules (i.e. rule-out malignancy), allowing physicians to safely recommend surveillance with a high negative predictive value (NPV) (Vargas-Salas *et al.* 2018). In addition, several of these tests have reported specificities as high as 80%, which result in positive predictive values comparable to the risk of a Bethesda V cytology (i.e. 60–70%) (Koster *et al.* 2018, Endo *et al.* 2019, Fazeli *et al.* 2020, Zafereo *et al.* 2020, Gortakowski *et al.* 2021).

ThyroidPrint^®^ is a novel q-PCR-based ten-gene classifier with its signature representing both the tumor microenvironment (CXCR3, CXCL10, CCR3, CCR7, and CXADR) and tumor epithelial cells (TIMP1, CLDN1, KTR19, AFAPL2, and HMOX1). The signature qPCR data is analyzed by a neural network algorithm that predicts the probability of an ITN of being benign (González *et al.* 2017). In a recent study reporting two-independent, multicenter clinical validation cohorts, ThyroidPrint^®^ predicted benign thyroid nodules with an NPV of 95%, while properly classifying 88% of benign cases (Zafereo *et al.* 2020). However, beyond the statistical performance, there are no post-validation studies reporting clinical utility (i.e. the impact on clinical decisions and net patient benefit), which is key to determine the clinical value of molecular testing for ITN. In this study, we report the first experience with ThyroidPrint^®^ to determine its clinical impact on surgical decisions and net patient benefit.

## Methods

### Study design

We performed a single-center, prospective, non-interventional study, collecting data between September 2018 and July 2021 (34 months). The study was approved by the ethics committee of the Pontificia Universidad Católica de Chile. All patients undergoing an FNA biopsy had a previous ultrasound revealing a thyroid nodule which the treating physician deemed appropriate for an FNA. The institutional protocol to decide the need for an FNA followed the 2015 ATA guidelines and ACR-TIRADS criteria. Upon initiation of the study, the ThyroidPrint^®^ test became commercially available to all physicians, who freely decided whether to perform molecular testing on patients with Bethesda III or IV category report. No other tests were offered to patients since these are not commercially available in the country. Physicians chose to use the ThyroidPrint^®^ test based on their own clinical judgment, ultrasound characteristics, cytology results, and patient preferences. Patients with a benign ThyroidPrint^®^ result were followed for a minimum of 12 months up to 34 months (interquartile range 12.2–18.9 months). Study endpoints included (i) benign call rate, (ii) positive predictive value for suspicious malignancy as per ThyroidPrint^®^ result, and (iii) rate of active surveillance.

### Patient recruitment, FNA sample collection, and follow-up

Inclusion criteria were as follows: 18-year-old or older with a thyroid nodule greater than 1.0 cm and less than 4.0 cm. Patients meeting inclusion criteria were eligible and invited to participate. Patients accepting to participate were asked to sign an informed consent. Collected data included demographics (age and gender) and relevant clinical data (pre-FNA ultrasound report and cytology). Only patients with a Bethesda category III or IV cytology report qualified to continue in the study. Follow-up was conducted by a designated nurse to review the electronic record of patients, including cytology result, molecular testing result (if performed), and final clinical decision, i.e. clinical surveillance vs surgery. In patients recommended for surveillance, follow-up was performed with a neck ultrasound every 6 months for the first year and every 12 months thereafter. To protect data privacy and avoid data loss, all data were collected prospectively and stored in a password-protected electronic data capture system. No patients categorized as Bethesda III or IV (with or without molecular testing) were lost to follow-up during this study.

The sample for molecular testing was collected at the time of the first FNA (performing two additional needle passes) or a new second FNA in patients with previous indeterminate cytology referred from outside the institution. Samples were collected in specially prepared cryovials with RNA-protected solution (Qiagen) and stored at −20℃ until a cytology report was available. All cytology findings were reported by an expert cytopathologist according to the Bethesda System for Reporting Thyroid Cytopathology (TBSRTC) (Faquin *et al.* 2017). Nodules reported as Bethesda III were sub-classified as atypia of undetermined significance (AUS) or follicular lesion of undetermined significance (FLUS), based on the presence or absence of nuclear atypia, respectively. Molecular testing was performed at the GeneproDx Clinical Laboratory (College of American Pathologist Accredited, number 1821095), Santiago, Chile.

### Ultrasound evaluation

All thyroid nodules were evaluated by following the ACR TI-RADS (Tessler *et al.* 2017) recommendations and classified into three risk groups: low (TI-RADS 1 to TI-RADS 3), intermediate (TI-RADS 4), and high (TI-RADS 5).

### Surgical management

For each patient, clinical management was conducted in a shared decision-making process including the attending physician, surgeon, and patient. In addition, the pros and cons of surgery or surveillance were discussed considering the patient’s personal preferences. For patients undergoing surgery due to a suspicious for malignancy as per ThyroidPrint^®^ result or who were recommended to directly undergo surgery without molecular testing, the extent of surgery (lobectomy or total thyroidectomy) was based on institutional practice following ATA guidelines. ThyroidPrint^®^ testing result was not used to guide the extent of surgery. In patients undergoing surgery, a diagram of the detailed location and size of the nodule of interest was provided by the surgeon to the pathologist to ensure correct matching of the indeterminate nodule to the pathology report. Surgical pathological diagnosis was reported by a board of certified expert head and neck pathologists (AS, PZ, and JCR). Resected nodules were classified as ‘nonsurgical’ to include all histologic subtypes that normally would not require surgery. Alternatively, resected nodules that were called malignant or non-invasive follicular thyroid neoplasm with papillary-like nuclear features were considered in the surgical disease group.

### Statistical analysis

Categorical variables were expressed as absolute numbers and percentages. Continuous variables were presented either as mean and s.d. or median and range. Categorical comparisons were performed with Chi-square test, and continuous variables were compared by using the Kruskal-Wallis H test. Multivariate regression analysis was conducted to identify potential variables that could individually affect results. A *P*-value of 0.05 or less was significant. Statistical analyses were performed using STATA v.16.0 (College Station, TX, USA: StataCorp LP).

## Results

### Thyroid nodules characteristics and tested patient profile

**Figure 1:**
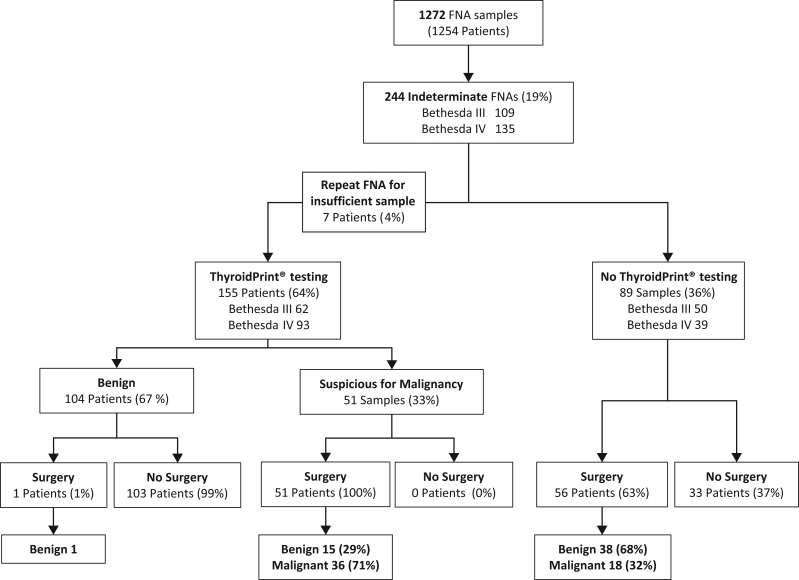
Patient medical decision and outcome diagram.

A total of 1272 FNA samples were collected from 1254 patients, of which 244 (19.2%) were reported as indeterminate: 109 Bethesda III (8.6%) and 135 Bethesda IV (10.6%) (Fig. 1). Physicians performed molecular testing in 155 (63.5%) of 244 indeterminate cases, of which 62 (40.0%) were Bethesda III and 93 (60.0%) Bethesda IV (Fig. 1). Seven patients (4.5%) underwent a second FNA due to an initially low RNA concentration, all of which yielded an appropriate sample in the second FNA (Fig. 1). As shown in Table 1, when comparing patients who underwent ThyroidPrint® with those who did not, there were no significant differences in age or sex, but tested patients had smaller nodules than non-tested (1.6 ± 0.1 cm vs 2.0 ± 0.2 cm, *P*-value < 0.05). No patients with nodules 4.0 cm or greater underwent testing.

**Table 1:** Demographics and thyroid nodule characteristics.

	No ThyroidPrint® testing		ThyroidPrint® testing		Total	
Total						
Physician recommendation	89	36%	155	64%	244	100%
Age						
Mean	54.4		49.9		51.6	
Range	23–82		19–81		19–82	
Sex						
Male	9	10%	24	15%	33	14%
Female	80	90%	131	85%	211	86%
Nodule size						
Median size (cm)	2.0		1.6^a^		1.7	
Range size (cm)	1.0–4.0		1.0–3.2		1.0–4.0	
1.0–1.99	61	69%	119	77%	180	82%
2.0–2.99	11	12%	32	21%^a^	43	20%
3.0–3.99	8	9%	4	3%^a^	12	5%
4.0	9	10%	0	0%^a^	9	4%
Cytological category						
Bethesda III	47	43%	62	57%^a^	109	45%
FLUS	30	56%	24	44%^a^	54	50%
AUS	17	31%	38	69%^a^	55	50%
Bethesda IV	42	31%	93	69%^a^	135	55%
ACR TI-RADS risk						
Low	23	26%	10	6%	33	14%
Intermediate	50	56%	136	88%^a^	186	76%
High	16	18%	9	6%	25	10%

^a^*P* < 0.05: ThyroidPrint® testing vs no ThyroidPrint® testing.

A total of 62 (56.9%) of 109 Bethesda III and 93 (68.9%) of 135 Bethesda IV patients underwent molecular testing. In patients with Bethesda III cytology, testing was performed more frequently in AUS than FLUS cases (69% vs 44%, respectively) (Table 1). Regarding ultrasound, an ACR-TIRADS intermediate risk was reported in 136 (87.7%) of 155 tested patients, compared with 50 (56.2%) of 89 patients who were not tested (*P* < 0.05) (Table 1). There were no significant differences in the rate of molecular testing between patients with low- and high-risk ACR-TIRADS ultrasound ACR-TIRADS (Table 1).

### Clinical decisions based on ThyroidPrint^®^ results

In tested patients, 104 of 155 were called benign (BCR of 67.1%) (Table 2). After a median follow-up of 15 months, only 1 (1.0%) of 104 patients with a benign ThyroidPrint^®^ result underwent surgery due to nodule growth with a final surgical biopsy reporting a follicular adenoma (Fig. 1, Table 2). The overall BCR for Bethesda III was significantly higher than for Bethesda IV cytology (76% vs 61%, *P* < 0.05) (Table 2). In patients with Bethesda III cytology, the BCR was significantly higher for FLUS compared to AUS (87% vs 58%, *P* < 0.05). Multivariate analysis including age, sex, nodule size, Bethesda category, ACR-TIRAD, and ThyroidPrint^®^ result showed that a benign ThyroidPrint^®^ result was the only independent factor to modify the clinical decision toward active surveillance, with a higher rate than in non-tested patients (67% vs 37%, respectively, *P* < 0.02). In patients without testing, the choice of surveillance was significantly higher for FLUS compared to AUS and Bethesda IV category (67% vs 11% and 32%, respectively, *P* < 0.05) (Table 2). All 51 patients with a suspicious for malignancy as per ThyroidPrint^®^ result underwent surgery, with a positive predictive value (PPV) higher than the PPV in non-tested patients (71% vs 32%, respectively, *P* < 0.05) (Tables 2 and 3). ThyroidPrint® decreased the rate of surgery from 63% (non-tested patients) to 33% (tested patients) (Table 2).

**Table 2:** ThyroidPrint^®^ results by Bethesda, ultrasound, and physician decision.

	No ThyroidPrint® testing				ThyroidPrint® testing			
	Active surveillance		Surgery		Benign		Suspicious of malignancy	
Patients	33	37%	56	63%	104	67%	51	33%
Cytology category								
Bethesda III	20	42%	28	58%	47	76%	15	24%^a^
FLUS	20	67%	10	33%^c^	33	87%	5	13%^b^
AUS	2	11%	16	89%	14	58%	10	42%^b^
Bethesda IV	13	32%	28	68%	57	61%	36	39%^a^
ACR TI-RADS risk								
Low	20	87%	3	13%	8	80%	2	20%
Intermediate	13	20%	37	80%	97	71%	39	29%^d^
High	0	0%	16	100%^b^	0	0%	9	100%
Histopathology								
Benign	N/A	–	38	68%	1	–	15	29%
Malignant	N/A	–	18	32%	N/A	–	36	71%^e^

^a^*P* < 0.05 Bethesda III vs Bethesda IV in patients undergoing ThyroidPrint® testing.

^b^*P* < 0.05 FLUS vs AUS in patients undergoing ThyroidPrint® testing.

^c^*P* < 0.05 surveillance for FLUS vs AUS and Bethesda IV in patient with no testing.

^d^*P* < 0.05 surveillance for intermediate TIRADS-ACR score no testing vs ThyroidPrint® testing.

^e^*P* < 0.05 Rate of malignancy for no testing vs ThyroidPrint® testing.

**Table 3:** Histopathological diagnosis in patients undergoing surgery.

	ThyroidPrint® testing		No ThyroidPrint® testing	
	Suspicious of malignancy		Surgery recommended	
Resected nodules	51	100%	56	100%
Non-surgical	15	29%	38	68%
Benign follicular nodule	4	27%	16	42%
Follicular adenoma	6	40%	11	29%
Follicular adenoma: oncocytic	4	27%	7	18%
Chronic thyroiditis	1	7%	4	11%
Other benign	0	0%	0	0%
Surgical (ROM)	36	71%	18	32%^a^
Malignant				
Papillary thyroid carcinoma				
Classic variant	7	19%	2	18%
Follicular variant	8	22%	5	27%
Tall cell variant	1	3%	1	5%
Follicular carcinoma				
Minimally invasive	4	11%	2	14%
Widely invasive	2	6%	1	0%
Oncocytic carcinoma	4	11%	3	14%
Poorly differentiated carcinoma	1	3%	0	0%
Medullary carcinoma	2	6%	0	0%
Others				
NIFTP	7	19%	4	23%

^a^ROM of suspicious of malignancy molecular testing call vs no testing.

NIFTP, non-invasive follicular thyroid neoplasm with papillary-like nuclear features; ROM, risk of malignancy.

### Surgical outcomes

Histopathological diagnoses for resected nodules are shown in Table 3. Overall, there was a similar frequency of histopathological subtypes for resected nodules for patients with a suspicious for malignancy as per ThyroidPrint^®^ result compared to non-tested patients undergoing diagnostic surgery (Table 3).

## Discussion

We report the first real-world experience for ThyroidPrint^®^, a ten-gene classifier intended to improve diagnostic accuracy of patients with ITN. The availability of molecular testing for patients with ITN outside of the United States is limited. ThyroidPrint^®^ has been recently validated in two independent multicenter clinical cohorts, showing equivalent performance in populations from the United States and South America, with an NPV of 95%, specificity of 88%, and PPV of 78% (Zafereo *et al.* 2020).

In this single-center, prospective, non-interventional study, physicians were free to offer molecular testing to patients with ITN, allowing us to assess the willingness to use this novel diagnostic tool, the impact on clinical decisions, and net patient benefit. Before ThyroidPrint^®^ became available at our institution, approximately 75% of ITN cases were recommended diagnostic surgery. A watchful waiting decision for the remaining 25% of patients was mainly recommended to patients with low ultrasound ACR-TIRADS risk findings and a Bethesda III FLUS cytology report with no nuclear atypia or a Bethesda II report in a repeat FNA. After ThyroidPrint^®^ availability, almost two-thirds of patients with indeterminate cytology were recommended molecular testing. Possible reasons that may have driven physicians not to recommend molecular testing were a low ACR-TIRADS risk and Bethesda III-FLUS lesions (in which surveillance is the preferred choice) (Valderrabano & McIver 2017) or alternatively a high risk ACR-TIRADS score or patient with nodules greater than 3 cm, leading to direct diagnostic surgery (Park *et al.* 2014, Valderrabano & McIver 2017). The BCR of 67% for ThyroidPrint^®^ resulted in a reduction of diagnostic surgeries. This result suggests that physicians are willing to recommend surveillance in cases with a benign result, providing initial evidence of the ThyroidPrint^®^ test clinical utility.

After a median 15-month follow-up, only 1% of patients with a benign ThyroidPrint^®^ result ultimately underwent surgery (with a benign final pathology). Although the final decision of recommending surgery relies on the physician’s judgment and patients’ preferences, it appears that physicians are comfortable recommending watchful waiting for a benign ThyroidPrint^®^ result.

Regarding the patient net benefit, ThyroidPrint^®^ resulted in a meaningful reduction of diagnostic surgeries of almost 50% (from 63% to 33%). Furthermore, testing resulted in a malignancy post-test probability of 71% for patients with a suspicious result, which is comparable with the malignancy risk of a Bethesda V cytology. This improvement in the PPV compared to that of diagnostic surgery (32%) may support the utility of ThyroidPrint^®^ as a rule-in test as well. Further sub-analysis suggests that the value of ThyroidPrint® testing is mainly in patients with intermediate-risk TI-RADS nodules, increasing the rate of active surveillance from 20% to 71%. However, this clinical benefit was not seen in patients with either low-risk or high-risk TI-RADS nodules, in which the rates of malignancy can be very low (5%) or very high (95%), respectively (Tessler *et al.* 2017, Valderrabano & McIver 2017, Rocha *et al.* 2018, 2019). Likewise, the presence of nuclear atypia in cytology increases the probability of malignancy up to 50%. These statistics suggest that molecular testing may not be necessary for all indeterminate cases and should be weighted with the previous results of the ultrasound and cytological report.

Importantly, based on the previously reported sensitivity of 91% (González *et al.* 2017, Zafereo *et al.* 2020), we can estimate that in this study, there were approximately four false-negative cases under surveillance that were not identified by molecular testing. Therefore, for this cohort, we can estimate a total of 40 cases with malignant nodules (36 true positives plus 4 estimated false negatives), which represents a disease prevalence of 26%, consistent with our previously determined institutional disease prevalence of 26.5% (Zafereo *et al.* 2020). Long-term follow-up should provide more clarity regarding the clinical relevance of this issue, particularly the incidence of malignant nodules among patients with a benign ThyroidPrint^®^ result. On the other hand, of 51 patients with a suspicious ThyroidPrint^®^ result, 36 proved to be malignant, yielding a PPV of 71%. This is consistent with the PPV of 78% previously reported in the clinical validation study (Zafereo *et al.* 2020).

During the last few years, several studies have evaluated the real-world performance of other commercially available molecular tests for ITN. Regarding Afirma GSC, the reported BCR varies from 61% to 78%, with associated surgery rates between 17.6% and 34.7% (Angell *et al.* 2019, Harrell *et al.* 2019, Martin *et al.* 2019, Wei *et al.* 2019). The dispersion of BCRs may be explained by the heterogeneity of the subjects included and the specific prevalence of malignancy in the different studies (ranging from 15% to 28%). For this reason, it is of utmost importance that every management team be aware of their institutional prevalence of malignancy for ITN when considering molecular testing. With respect to Thyroseq v3, a recent study from Desai *et al.* reported a BCR of 71% and a surgery rate of 31% (Desai *et al.* 2021). This data, although similar to our results, is not fully comparable because their series lacked a non-tested group and not all the patients with a suspicious Thyroseq v3 underwent surgery.

A major strength of this study was the inclusion of a non-tested group of patients with ITN. This allowed a more reliable analysis of the impact of ThyroidPrint^®^ regarding the reduction of diagnostic surgery and patient net benefit. However, this study has several limitations. First, it is a single-center study in a tertiary reference academic institution which may not be representative of the routine community practice. Therefore, new independent post-validation studies are needed to further provide evidence of clinical utility. Second, a longer follow-up timeframe is necessary to accurately determine the net benefit for patients (true long-term avoidance of surgery) and cost-effectiveness. Third, longer follow-up of tested nodules reported as benign that did not undergo surgery is required to better determine the false-negative rate.

Finally, the lack of experience of physicians with genomic testing and patient anxiety may have diminished the willingness to choose molecular testing.

In summary, this first real-world experience supports the clinical utility of ThyroidPrint^®^ for ITN, showing the willingness of physicians to offer molecular testing as well as the change in clinical management based on the test result. The 48% reduction in diagnostic surgery shows ThyroidPrint^®^ testing may provide benefits to a meaningful number of patients. New independent studies will be key to provide additional evidence supporting the use of molecular testing in ITN as part of routine clinical practice.

## Declaration of interest

All authors are employees of the Pontificia Universidad Católica de Chile (PUC). The Pontificia Universidad Católica de Chile has granted GeneproDx a license to market ThyroidPrint for commercial use. They receive no compensation, directly or indirectly, related to GeneproDx. Internal resources from the departments of endocrinology and surgical oncology were used to execute this study.

## Funding

No external funding was received to perform this study.
